# Aroma compounds and physicochemical and functional properties of traditional Tokat bread

**DOI:** 10.1002/fsn3.4468

**Published:** 2024-10-23

**Authors:** Ali Cingöz

**Affiliations:** ^1^ Department of Food Engineering Tokat Gaziosmanpasa University Tokat Turkey

**Keywords:** functional properties, pGI, texture, volatile compounds, yeast isolation

## Abstract

This study aimed to determine the physical (height, weight, volume, color, etc.), chemical (protein, fat, ash, moisture, etc.), functional (total phenolic, flavonoid, and antioxidant capacity), nutritional (total, soluble, insoluble dietary fiber, and important starch fractions), and texture properties and volatile organic compounds of traditional Tokat bread. In addition, yeast and bacterial species were isolated and identified from the sourdoughs used in its production. Having a 6‐day shelf life, traditional Tokat bread has an average total dietary fiber content of 14.64%. The predictable glycemic index was 83.09, and the slowly digestible starch content was 6.15%. In traditional Tokat bread, 45 different volatile aroma components were identified, and *S*. *cerevisiae*, *D*. *hansenii*, *K*. *lactis*, *L*. *plantarum*, and *L*. *mesentorides* species were mostly isolated in sourdough. The characteristics of traditional Tokat breads produced by traditional methods in different regions were determined. It is predicted that the taste and aroma of traditional Tokat bread originate from the hornbeam and pelite woods used in the baking stage and the sourdough used. The yeast composition of the breads collected from different regions also varies.

## Graphical abstract

In traditional Tokat bread, 45 different volatile aroma components were identified and *S*. *cerevisiae*, *D*. *hansenii*, *K*. *lactis*, *L*. *plantarum*, and *L*. *mesentorides* species were mostly isolated in sourdough. It is predicted that the taste and aroma of traditional Tokat bread originate from the hornbeam and pelite woods used in the baking stage and the sourdough used. The predictable glycemic index was 83.09, and the slowly digestible starch content was 6.15%.

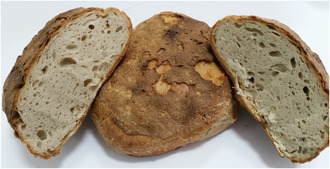

## 1 INTRODUCTION

Bread is a foodstuff widely produced in different yeast and unleavened types and in different ways. There are many commercial and traditionally produced bread varieties, an indispensable food for Turkish people (Meral et al., 2023). Turkish bread can be classified into four groups according to flour type (wheat, corn, etc.), baking method (oven, stone, tandoor, etc.), shape (loaf, pan, flat, etc.), and yeast and unleavened (Koca & Yazıcı, 2014). Currently, commercial yeasts are the most favored method for industrial bread production, although some traditional bakeries use sourdoughs that have been produced for many years (Siepmann et al., 2018). Bread production by sourdough fermentation is known as a biotechnological process that is applied worldwide and has a long history (Vuyst et al., 2017). It has a special ecosystem that produces bakery products and contains lactic acid bacteria (LAB) and yeasts. LAB affects acidification, LAB and yeasts affect flavor formation, and yeasts and heterofermentative LAB affect fermentation (Vuyst et al., 2017). The use of sourdough yeast in bread making positively affects the sensory properties of bread and extends the physical and microbiological shelf life.

Sourdough is a product obtained by the growth of lactic acid bacteria and yeasts. Lactic acid bacteria produce organic acids, CO_2_ (usually heterofermentative bacteria), exopolysaccharides, antifungals, and bacteriocins, while yeasts produce ethyl alcohol and CO_2_. The organic acids produced promote gluten proteolysis (amino acids involved in Maillard reactions), starch hydrolysis, and lipid oxidation (release of volatile components). In sourdough production, aroma formation is caused by organic acids and ethyl alcohol produced by yeasts (Siepmann et al., 2018). There are four types of sourdough processes in bread production technology. Type I sourdough is a traditional process using a portion of the mature sourdough produced in the previous fermentation (Sağdıç et al., 2021). Type II sourdough is a type of sourdough in liquid form obtained using a starter culture. It is mostly used in industrial production. It is produced by adding LAB and yeasts adapted to the environment to the dough at a ratio of 100:1. Type III sourdough is obtained by drying type II sourdough in liquid form using various drying techniques (Sağdıç et al., 2021). In type III sourdough, mixed cultures of unleavened Lb. *brevis*, Lb. *plantarum*, and Pc. *pentosaceous* are more commonly identified (Liu, 2014). Type IV sourdough is used in traditional bakeries and laboratory studies. Sourdough prepared using starter culture is reproduced by the renewal technique in the traditional method (Sağdıç et al., 2021). Type IV is a mixture of type I and type II sourdough produced on a laboratory scale (Siepmann et al., 2018).

**FIGURE 1:**
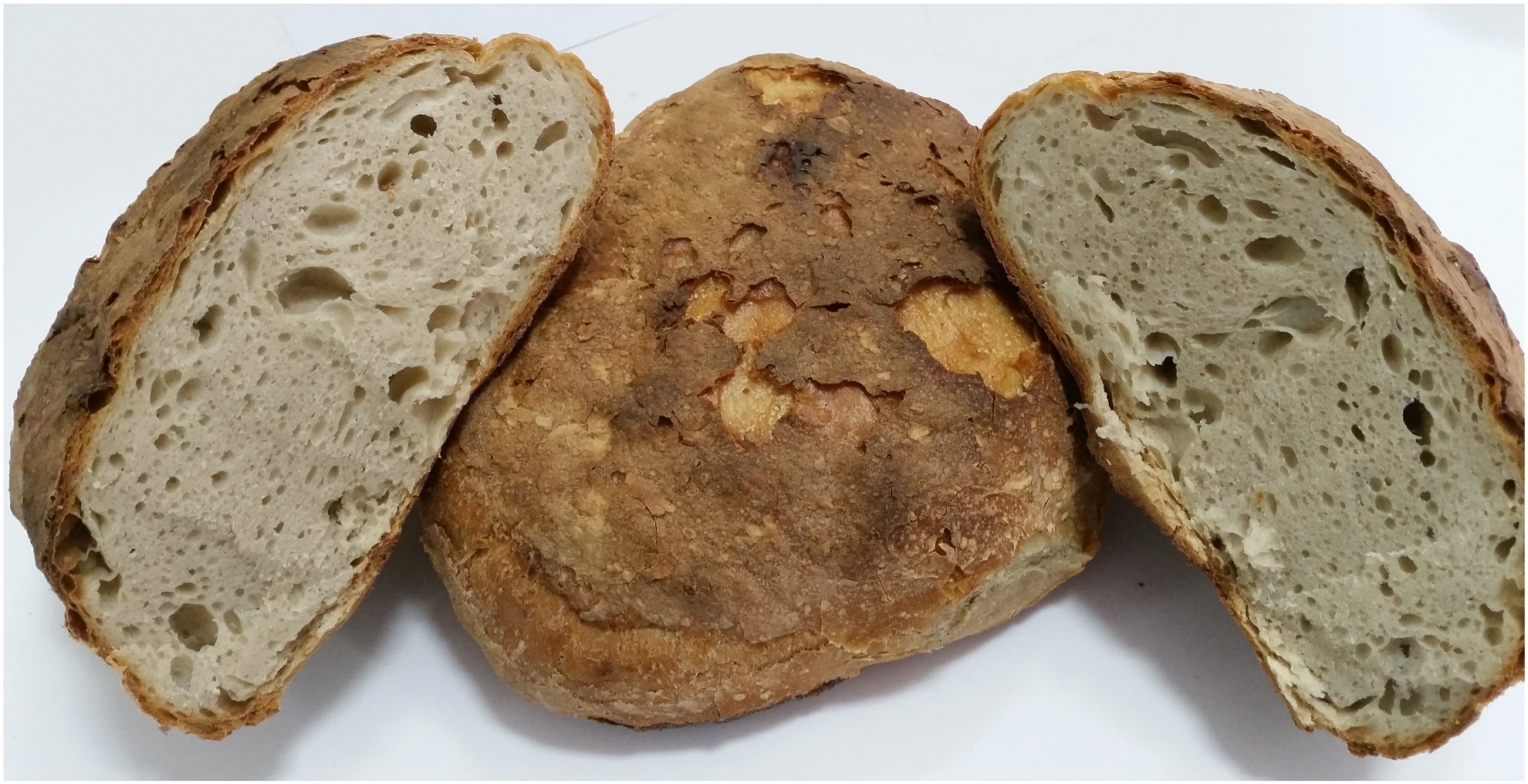
Traditional Tokat bread and its crust structure.

Traditional Tokat bread is a traditional bread produced with the type I sourdough process and stands out with its flavorful structure and long shelf life. It is well baked and puffy, homogeneous in the distribution of crust color, spongy inside when cut, and without crust‐inside separation. Figure 1 shows Tokat traditional bread and its internal structure. It is made from whole wheat flour (100 kg), water (30 L), sourdough yeast (25 kg), and salt (1 kg). The dough is kneaded for 45–60 min at 25–30°C until the homogenous, elastic, and viscous properties are obtained. As a result of the kneading process, the dough is desired to have a shiny appearance, be soft, smooth, and slippery. The resulting dough is left for 90 min at room temperature for mass fermentation. At this stage, the volume of the dough increases by 30%–40% compared to its initial volume. Approximately 1700–1800 g of dough is taken from the fermented dough by hand (the weight of the dough is not weighed; it is weighed by the rule of thumb and placed on the oven shovel and placed in the oven). The bread is baked in special ovens at 200–220°C for 90 min and cooled for 20–30 min without overlapping and then sent for sale.

The ovens used for baking traditional Tokat bread are specific to the geographical boundary. The floor is 1 m high in sand, 3 tons of baked rock salt is laid on top of it, fire bricks are laid on top of the salt, and the side walls are built with whole bricks. For the heating of the furnace, firewood such as pelite (*Quercus petraea* Lieb.) (90%) and hornbeam (*Fagus orientalis* Lipsky) (10%) wood, which have plenty of flame and heat, are used. The incense and resinous compounds from the fire of these firewoods give traditional Tokat bread its unique flavor.

There are many studies on traditional breads in the world. In recent years, studies in which aroma compounds of traditional breads are determined and yeast isolation studies have gained weight. In this context, aromatic hydrocarbons were detected in traditional Iranian breads (Jahed Khaniki et al., 2021). Volatile and flavor compounds were determined in steamed Chinese breads (Wang et al., 2021). Flavor compounds were identified in sourdough Italian bread (Gaglio et al., 2023). The dynamics of volatile compounds in triticale bread produced with sourdough were determined (Galoburda et al., 2020). Lactic acid bacteria and yeasts were characterized in sourdoughs used in Japanese bakeries (Fujimoto et al., 2019). Different studies are carried out to standardize the breads produced by traditional methods and transfer them to future generations. There are studies on yeast isolation and identification in breads produced with local sourdoughs, such as traditional Afyonkarahisar bread (Denizkara, 2021), Trabzon Vakfıkebir bread, Akhisar home bread, Gerede home bread (Solak, 2020), determination of color and texture properties of Karahöyük Zığır bread (Demiray et al., 2015), and comparison of volatile component profiles of sourdough breads produced in İzmir province (Bakırcı, 2019).

This study aimed to standardize the production of Tokat traditional bread, traditionally produced in Tokat province and having Mahreç Mark (No. 896), and to determine its unique properties. For this purpose, physicochemical (volume, weight, height, specific volume, color, moisture, ash, protein, and fat content, etc.), functional (total phenolic and flavonoid substances, antioxidant activity, important starch fractions, dietary fiber, etc.), flavor compounds, and sourdough identification and characterization were determined.

## 2 MATERIALS AND METHODS

### 2.1 Materials

Traditional Tokat bread was obtained from 5 different local producers in Tokat, Turkey and stored in room conditions. Total dietary fiber kit (K‐TDFR), amyloglycosidase (3300 U/mL) from Megazyme (Ireland), glucose oxidase peroxidase (GOPOD) kit from Biasis (Turkey), pancreatin (Porcine pancreas 4 × USP), invertase (3000 EU/ml), ABTS, TPTZ, DPPH, Trolox, MESTRIS, quercetin, caffeic acid, 4‐hydroxybenzoic acid, vanilic acid, ferulic acid, 4‐hydroxy‐3,5 dimethoxy benzoic acid, acetic acid, uronic acid, and furfural were purchased from Sigma‐Aldrich (St. Louis, MO, USA). Folin–Ciocalteu reagent, iron(III) chloride hexahydrate, potassium persulfate, D(+)glucose, D(+)xylose from Merck (Germany), 3,4,5‐trihydroxy benzoic acid (gallic acid), and Ankom XT4 cartridges from Ankom Technology (USA). GC–MS analysis column, DB‐5MS capillary column (60 m × 0.25 mm × 0.25 μm) was provided J&W Scientific (USA), DB‐WAX capillary column (60 m × 0.25 mm × 0.25 μm) was provided J&W Scientific (USA). Other chemicals used are of analytical standard Sigma (USA) and Merck (Germany) were obtained from the companies.

### 2.2 Physical analysis

Physical analyses were carried out on 50 breads produced by five different local producers. The weights (g) and heights (cm) of all the bread were measured. The crust thickness was measured in mm with the help of calipers from at least 3 different points after the breads were cut in half. Their volumes were determined using the rapeseed displacement method according to AACC Standard Method No. 10‐05.01 (AACC International, 2004). The specific volume of the bread was calculated as the ratio between the volume (cm^3^) and the weight (g) of the bread (Araki et al., 2009).

Specific volume = volume (cm^3^)/weight (g).

The color of the crumb and crust of the bread samples was measured with a Minolta CR300 (Minolta Inc., Tokyo, Japan) using the Hunter L*, a*, and b* color scales.

### 2.3 Chemical analysis

Chemical analyses were carried out by taking 5 samples from each establishment, and the results were averaged. The moisture and ash content of the breads were determined according to AACC Standard Method No. 44‐01.01 and 08‐01.01, respectively (AACC International, 2004). The micro‐Kjeldahl method was used to determine nitrogen content (nitrogen factor 5.70) (AOAC, 2000). Crude fat was determined gravimetrically in an Ankom XT10 extraction system using the Filter Bag XT4 technique after petroleum ether extraction of the dried sample (Ankom Technology Inc., Macedon, NY) (AOCS, 2005). Water activity was measured using an AquaLab (Model Series 3TE) water activity device set at 20°C (Hughes et al., 2002). Titratable acidity was determined according to AOAC. The titratable acidity was calculated as percent lactic acid.

### 2.4 Texture profile analysis

The texture measurements of the breads were performed (Vtest: 35 mm/min, Vreturn: 500 mm/min, Vpos_1_: 500 mm/min, Vpos_2_: 10 mm/min, Lmax: 10 mm, Fv: 0.1 N) for 8 days. The breads were kept in closed polythene containers under room conditions for 8 days. The hardness measurements of the breads were determined in Newton/cm^2^ according to Aydin (1991) using a texture analyzer (a round head of 2 cm diameter and at a pressure of 10 mm) (Zwick Z0.5, Germany) at different time intervals.

### 2.5 Phenolic and flavonoid substances and antioxidant capacity analysis

The collected breads were subjected to an extraction procedure to determine the total phenolic and flavonoid substances. The extraction was carried out by mixing (200 rpm, 1 h, room temperature) 25 g of sample with 50 mL of 50% acetone. The extract was separated by filtration, and this procedure was repeated 3 times. All the extracts were combined, centrifuged at 2500×g for 10 min (Boeco U‐32R, Germany), and evaporated at 45°C until around 10 mL of supernatant was obtained. The volume of final solutions was made up to 25 mL with distilled water and stored at −18°C until use (Eberhardt et al., 2000).

The phenolic substance of the extracts was measured by the Folin–Ciocalteu method using gallic acid, kafeic acid, 4‐hydroxybenzoic acid, vanillic acid, trans ferulic acid, and 4‐hydroxy‐3,5 dimethoxy benzoic acid as a standard (Singleton & Rossi, 1965). Their total flavonoid substance was measured by Li et al. (2015) using the method with quercetin standards. The antioxidant capacities of the extracts were measured by 2,2 diphenyl‐1‐picrylhydrazyl radical scavenging activity (DPPH) (Brand‐Williams et al., 1995), iron (III)‐reducing antioxidant power (FRAP) (Benzie & Strain, 1996), and trolox equivalent (TE) antioxidant capacity (ABTS) (Re et al., 1999) using Trolox as standard.

### 2.6 Determination of important starch fractions

Total glucose (TG), rapidly available glucose (RAG), total starch (TS), rapidly digestible starch (RDS), slowly digestible starch (SDS), free glucose (FG), and starch hydrolysis index (SHI) of breads were determined by the in vitro digestion method (Englyst et al., 1992). The ground samples, 1.00 ± 0.01 g, were placed in 15 mL glass tubes, and 50 mg of guar gum, 15 pieces of 4 mm glass beads, and 4 mL of acetate buffer (0.5 M, pH 5.2, and 5 mM CaCl_2_) were added to the tubes and vortexed for 30 s. To the mixture, 1 mL of the hydrolytic enzyme mixture (0.90 g of pancreatin‐Sigma P7545) was dissolved in 4 mL of water and mixed in a magnetic stirrer for 10 min. Centrifugation was then performed at 1500×*g* for 10 min, and the supernatant was separated. To the mixture, 2.7 mL of the pancreatin supernatant, 0.3 mL of amyloglucosidase (0.32 mL of amyloglucosidase was diluted with 0.4 mL of distilled water) and 0.2 mL of invertase (10 mg/mL) were added and mixed by vortexing and shaken in a water bath at 37°C in a horizontal position at 160 rpm. After 20 (G20) and 120 (G120) minutes, 0.2 mL of the sample was removed from the tubes and transferred to tubes containing 4 mL of 95% ethanol to stop the reaction (G20, G120). G20 and G120 fractions were centrifuged at 4000×g for 10 min (Boeco U‐32R, Germany), and glucose amounts in their supernatants were determined by the glucose oxidase‐peroxidase (GOPOD) method. The significant starch fractions were calculated with the following equations.

TS = (TG − FG) × 0.9.

RAG = G_20_.

RDS = (G_20_ − FG) × 0.9.

SDS = (G_120_ − G_20_) × 0.9.

SHI = (RDS/TS) × 100.

### 2.7 Predicted glycemic index

The hydrolysis index (HI) was determined by dividing the area under the hydrolysis curve of the sample by the area under the standard glucose curve. The predicted glycemic index (pGI) of the samples was calculated according to the following equation (Chung et al., 2008):

pGI = 39.71 + 0.549 HI.

### 2.8 Dietary fiber analysis

Total dietary fiber (TDF), soluble dietary fiber (SDF), and insoluble dietary fiber (ISDF) contents in bread were determined according to standard methods AOAC‐991.43 (AOAC, 2000) and AACC‐32‐07 (AACC International, 2004) by using the Total Dietary Fiber Assay Kit (Megazyme K‐TDFR). To 1.000 ± 0.005 g of the ground samples, 40 mL of MES‐TRIS buffer (pH 8.2) was added. The sample was treated in order with 50 μL of heat‐stable α‐amylase (3000 U/mL, 30 min, 98–100°C), 100 μL of protease (350 tyrosine U/ml, 30 min, 60°C), and 200 μL of amyloglucosidase (3300 U/mL, 30 min, 60°C, pH 4.1–4.8). The mixture was filtered through Celite 545, dried goach crucibles (40–60 μm pores), and washed twice (10 mL 70°C distilled water, 10 mL 95% ethanol, 10 mL acetone). After that, the crucibles were dried in an oven at 103°C to a constant weight and weighed to determine the insoluble dietary fiber. The filtrate was used for the determination of soluble dietary fiber. To the filtrates, 4 volumes of 95% ethanol were added, kept at room temperature for 1 h to form a precipitate, and filtered through Celite 545. The residue remaining in the crucibles was washed in order with 78% ethanol and 95% ethanol and dried in an oven at 103°C to a constant weight to determine the soluble fiber. Total dietary fiber was calculated using the following equation:

$$\text{Dietary fiber}=\frac{R-p-A-B}{m}\times 100$$

m = sample weight, R = residue weight from m, A = ash weight from R, B = blank, p = protein weight from R.

### 2.9 Determination of the volatile compounds

Identification and quantification of volatile organic compounds in the flavor of traditional Tokat bread were carried out by modifying the method reported by Wang et al. (2021). Extracts were obtained from breads by the method reported by Galoburda et al. (2020). Compound identification was performed using the Adams mass spectral database (Adams, 2017), NIST 11, Wiley 9, and FFNSC 2.

### 2.10 Isolation of LAB and yeasts from sourdough

In the study, 20 sourdough samples taken from 5 different local bakeries in Tokat under aseptic conditions and stored at 4°C were used, and yeast and LAB analyses of the samples were performed. Isolation of LAB and yeast from the samples was carried out by the methods recommended by Fujimoto et al. (2019). The samples were weighed at 25 g under aseptic conditions and homogenized with a stomacher for 2 min using 225 mL of 1% (w/v) peptone solution for yeast analysis and 225 mL of buffered peptone water for LAB analysis. Successive dilutions were prepared from the homogenate with 1% peptone water and inoculated into the following media. According to the relevant methods, for yeast analyses, the samples were inoculated on Dichloran Rose Bengal Chloramphenicol (DRBC) agar by smear method because it contains less than 0.95% water activity. For LAB analyses, the samples were inoculated on Lactobacillus Agar acc. DE MAN, ROGOSA, and SHARPE (MRS) agar by the pouring method in two parallel cultures. The inoculated media were incubated at 30°C for 72 h and at 25°C for 5 days for LAB and yeast growth, respectively. Isolates obtained from typical colonies growing on the media were then subjected to diagnostic tests. Gram staining and catalase tests were performed to identify selected yeast and LAB isolates, and species identification and confirmation of the isolates at the biochemical and molecular level were performed by Vitek 2 biochemical and real‐time PCR methods. At the end of the incubation period, two or more representative colonies were selected from the typical yeast and LAB colonies growing on the medium. The selected different yeast and LAB colonies were purified by growing on a general medium Potato Dextrose Agar at 25°C for 2 days and Nutrient Agar at 30°C for 3 days, respectively. The purified isolates were identified by VITEK® 2 Compact (Biomeriux, France) according to their biochemical characteristics. VITEK®2 gram‐positive identification card (GP) was used for lactic acid bacteria, and VITEK®2 yeast identification card (YST) was used for yeast species. DNA isolation of yeast and LAB isolates from the identified species was carried out according to the innuPREP Bacteria DNA Kit method. Specific primers and probes were designed for the ITS1 gene region for LAB species, and the 23S rRNA gene region for yeast species, and the isolated DNAs were identified at the molecular level by real‐time PCR (Stratagene‐Agilent, Agilent, USA).

### 2.11 Statistical analysis

The SPSS statistical program (SPSS, Inc., Chicago, IL, USA) was used; an analysis of variance of the results (ANOVA) was performed, and the differences between groups were statistically evaluated with a 95% confidence interval by the Duncan multiple comparison test.

## 3 RESULTS AND DISCUSSION

A total of 50 traditional Tokat bread were collected from 5 different regions, and the results of the physical analysis of the breads were given in Table 1. Traditional Tokat bread, popularly consumed by the local people in the region, does not have a standard grammage. Since in the production, weighing of dough is done according to the rule of thumb of the producers, the standard deviations in the physical properties of the breads are high. The traditional Tokat bread type stands out with its large size, and the average weight of the samples collected was 1609.75 g and the average volume was 3641.36 cm^3^. It had a thick and hard crust structure, the average crust thickness was 6.79 mm, and the crust/crumb ratio was 43%. Since it is baked in wood‐fired stone ovens for a long time, it has a dark red/brown crust. The crust L* value of the bread samples was 46.77, a* value 8.73, and b* value 25.08, while the crumb L* value was 63.19, a* value −1.24, and b* value 13.91. The crust and crumb images of the breads were shown in Figure 1.

**TABLE 1:** Physical analysis results of breads.

Parameters	
Weight (g)	1609.75 ± 134.21
Height (cm)	12.46 ± 1.84
Volume (cm^3^)	3641.36 ± 301.10
Specific volume (cm^3^/g)	2.26 ± 0.09
Crust/Crumb (%)	43.00 ± 1.14
Crust thickness (mm)	6.79 ± 0.30
Crust color	
L*	46.77 ± 1.39
a*	8.73 ± 0.68
b*	25.08 ± 1.47
Crumb color	
L*	63.19 ± 0.51
a*	−1.24 ± 0.10
b*	13.91 ± 0.32

The breads collected were coded as TM1, TM2, E1, K1, and N1. Chemical analysis results of traditional Tokat breads are given in Table 2. The moisture content of the breads varied between 39.04 and 43.06%. Due to the flour with high ash content used in its production, it has a higher protein content than Turkish‐type francala breads (10.85 ± 0.40%). The protein content of traditional Tokat breads varies between 11.48 and 12.45%. Ash content ranged between 1.47 and 1.53%, but no statistically significant difference was detected. While the fat content of the breads varied between 1.21 and 1.42%, the enterprise‐coded E1 was statistically different in terms of fat content (*p* < .05). Water activity (aw) is an important parameter in the prevention of microbial growth (Tapia et al., 2020). The minimum water activity value for many spoilage yeast strains to thrive is 0.88 (Jdaini et al., 2022). The water activity value of the breads was determined in the range of 0.86–0.88. There was no statistical difference between the water activity values of the breads. Titratable acidity values of the breads in terms of lactic acid were determined between 3.98 and 4.16%.

**TABLE 2:** Chemical analysis results of breads.

	TM1	TM2	E1	K1	N1
Moisture (%)	41.39 ± 1.71^b^	39.04 ± 0.89^c^	43.06 ± 0.45^a^	42.08 ± 1.02^ab^	41.11 ± 0.78^b^
Protein (%)	11.90 ± 0.11^bc^	12.16 ± 0.23^ab^	11.48 ± 0.16^c^	12.45 ± 0.32^a^	12.05 ± 0.08^b^
Fat (%)	1.42 ± 0.03^a^	1.39 ± 0.02^ab^	1.21 ± 0.06^c^	1.38 ± 0.12^ab^	1.40 ± 0.04^a^
Ash (%)	1.50 ± 0.03^a^	1.47 ± 0.04^b^	1.49 ± 0.01^b^	1.53 ± 0.12^a^	1.51 ± 0.06^a^
Water activity (a_w_)	0.88 ± 0.02^a^	0.86 ± 0.02^a^	0.88 ± 0.03^a^	0.87 ± 0.01^a^	0.87 ± 0.03^a^
Titratable acidity (%)	4.02 ± 0.14^b^	3.98 ± 0.02^b^	4.16 ± 0.06^a^	4.04 ± 0.11^ab^	3.99 ± 0.12^b^

*Note*: The different letters indicate statistical differences at the *p* < .05 level of the samples in the same line.

The changing living conditions and increases in health problems lead consumers to healthier, more nutritious, and functional foods. In this context, consumers prefer products with high antioxidant activity and dietary fiber and without additives. The total phenolic and flavonoid substances and total antioxidant capacity of traditional Tokat breads are shown in Table 3. Total phenolic substance was determined using 6 different standards (Gallic Acid, Caffeic Acid, 4‐Hydroxybenzoic Acid, Vanillic Acid, Trans Ferulic Acid, 4‐Hydroxy‐3,5 Dimethoxy Benzoic Acid). Phenolic acids and flavonoids represent whole grains' most common form of phenolic compounds. They are among the main and most complex groups of phytochemicals, with a number of species existing as soluble free compounds, insoluble, and bound forms (Verardo et al., 2018). Gallic acid is widely used in the determination of total phenolics of various plant materials (Bastola et al., 2017). Ferulic acid is the most abundant simple phenolic acid in wheat (Adom et al., 2003). Syringic, p‐coumaric, p‐hydroxybenzoic, and vanillic acids are also found in significant amounts in wheat (Fardet, 2010). Differences in bread‐making processes have been reported to affect the phenolic profiles of flours significantly (Tian et al., 2021). Therefore, in the analysis of phenolic substances in breads, evaluation only from gallic acid equivalents may be insufficient. Total phenolic contents of traditional Tokat breads were determined to be 102.1–111.6 mg GAE/100 g, 48.8–51.1 mg CAE/100 g, 147.8–151.3 mg HAE/100 g, 102.0–108.6 mg VAE/100 g, 99.8–105.6 mg TFAE/100 g, and 158.5–162.6 mg DBAE/100 g, respectively. Patil et al. (2016) determined the total phenolic content of the bread produced with 100 g wheat flour as 20.83 mg GE/100 g and 29.59–80.74 mg GE/100 g in breads produced by adding millet at 3 different ratios (10%, 20%, and 30%). Since antioxidant capacity is affected by many factors, more than one method should be used for its evaluation (Song et al., 2010). The antioxidant activity of the breads was determined with three different methods, and they were found to have antioxidant activity in the range of 1.6–2.4 μM TE/100 g by the DPPH method, 9.1–12.1 μM TE/100 g by the ABTS method, and 5.1–6.6 μM TE/100 g by the FRAP method. Liu et al. (2010) determined the antioxidant activity of DPPH in six different wheat breads ranging from 6.48 to 8.57 μmol TE/g. Sourdough fermentations have been shown to increase antioxidant activity, inhibit lipid oxidation, and reduce the content of antinutritional factors. It is also reported that some of the exopolysaccharides produced by LAB have prebiotic properties (Suchintita et al., 2023). Although the total flavonoid substance of the breads varied between 21.4 and 25.1 μg QE/100 g, there were no statistically significant differences. Chlopicka et al. (2012) showed the total flavonoid of bread from wheat flour as 20.30 μg catechin per gram. Total flavonoid content is similar to the literature.

**TABLE 3:** Functional analysis results of breads.

	TM1	TM2	E1	K1	N1
Total phenolic substance (mg GAE/100 g)	106.4 ± 11.7^b^	102.1 ± 9.4^bc^	111.6 ± 4.2^a^	108.3 ± 8.7^ab^	106.5 ± 10.2^b^
Total phenolic substance (mg CAE/100 g)	48.8 ± 1.2^bc^	51.1 ± 1.4^a^	49.6 ± 1.3^bc^	49.9 ± 1.2^b^	50.4 ± 0.9^ab^
Total phenolic substance (mg HAE/100 g)	147.8 ± 6.4^b^	150.1 ± 3.1^a^	149.5 ± 4.3^ab^	149.8 ± 6.2^ab^	151.3 ± 5.8^a^
Total phenolic substance (mg VAE/100 g)	106.4 ± 4.5^ab^	104.4 ± 2.8^b^	108.6 ± 3.2^a^	102.0 ± 4.1^bc^	107.3 ± 2.9^a^
Total phenolic substance (mg TFAE/100 g)	105.3 ± 4.5^a^	99.8 ± 4.2^c^	102.5 ± 3.3^b^	104.1 ± 1.8^a^	105.6 ± 2.7^a^
Total phenolic substance (mg DBAE/100 g)	158.5 ± 6.4^b^	160.1 ± 2.6^a^	159.7 ± 3.6^ab^	162.6 ± 3.0^a^	161.8 ± 4.1^a^
DPPH (μM TE/100 g)	1.6 ± 0.3^c^	2.2 ± 0.5^a^	1.8 ± 0.6^b^	1.9 ± 0.4^b^	2.4 ± 0.1^a^
ABTS (μM TE/100 g)	9.1 ± 0.8^b^	10.6 ± 1.1^ab^	9.8 ± 1.3^b^	12.1 ± 0.8^a^	11.6 ± 0.6^a^
FRAP (μM TE/100 g)	5.5 ± 0.4^c^	5.1 ± 0.2^c^	6.6 ± 0.3^a^	6.4 ± 0.1^a^	5.9 ± 0.3^b^
Total flavonoid substance (μg QE/100 g)	23.5 ± 2.2^a^	25.1 ± 1.8^a^	21.4 ± 2.2^b^	22.8 ± 1.4^b^	24.6 ± 2.0^a^

*Note*: The different letters indicate statistical differences at the *p* < .05 level of the samples in the same line.

Abbreviations: DBAE, 4‐Hydroxy‐3,5 Dimethoxy Benzoic Acid Equivalent; GAE, Gallic Acid Equivalent; HAE, 4‐Hydroxybenzoic Acid Equivalent; KAE, Caffeic Acid Equivalent; TFAE, Trans Ferulic Acid Equivalent; VAE, Vanillic Acid Equivalent.

Total, soluble, and insoluble dietary fiber values of the breads are shown in Table 4. Soluble and insoluble dietary fibers reduce the risk of certain diseases and various types of cancer. The total dietary fiber content of traditional Tokat breads, which stand out with their high dietary fiber content, was determined between 14.03% and 15.07%, soluble dietary fiber content between 2.04% and 3.01%, and insoluble dietary fiber content between 11.92% and 12.98%.

**TABLE 4:** Important starch fractions and dietary fiber results of breads.

	TM1	TM2	E1	K1	N1
Total dietary fiber (%)	14.03 ± 0.28^c^	15.07 ± 0.41^a^	14.64 ± 0.16^b^	15.02 ± 0.22^a^	14.44 ± 0.31^b^
Soluble dietary fiber (%)	2.11 ± 0.11^c^	3.01 ± 0.08^a^	2.52 ± 0.13^b^	2.04 ± 0.22^c^	2.30 ± 0.18^bc^
Insoluble dietary fiber (%)	11.92 ± 0.18^bc^	12.06 ± 0.05^b^	12.12 ± 0.11^b^	12.98 ± 0.06^a^	12.14 ± 0.14^b^
RAG (%)	34.16 ± 0.12^b^	33.20 ± 0.16^c^	35.04 ± 0.23^a^	34.18 ± 0.08^b^	33.64 ± 0.45^c^
RDS (%)	30.13 ± 0.14^b^	29.88 ± 0.06^c^	29.12 ± 0.17^bc^	31.32 ± 0.24^a^	30.48 ± 0.14^b^
SDS (%)	5.98 ± 0.23^b^	6.05 ± 0.12^b^	6.33 ± 0.08^a^	6.24 ± 0.21^a^	6.16 ± 0.18^ab^
SHI	78.16 ± 1.02^b^	79.20 ± 0.98^a^	78.66 ± 1.04^ab^	80.01 ± 1.45^a^	79.14 ± 0.87^a^
pGI	82.62	83.19	82.89	83.63	83.15

*Note*: The different letters indicate statistical differences at the *p* < .05 level of the samples in the same line.

There are several problems (such as obesity, diabetes, and cardiovascular disease) with excessive bread consumption due to its starch content. Starch, which occupies an important place in the human diet, is classified on the basis of its in vitro digestion rate and speed into rapidly digestible starch (RDS), slowly digestible starch (SDS), and resistant starch (RS) (Dona et al., 2010). The nutritionally important starch fractions of the bread samples were determined in vitro, and the results are shown in Table 4. Consumption of foods with a high content of rapidly available glucose (RAG) causes a sudden increase in blood glucose levels, disrupting the sugar regulation of metabolism (Ergun, 2014). Foods with a low glycemic index have lower RAG values. The RAG content, which was 33.20%–35.04% in the traditional Tokat breads. In the context of prevention and control of metabolic diseases, it is important to choose foods with a high content of slowly digestible starch (SDS) and a low content of rapidly digestible starch (RDS) (Venn & Mann, 2004). The RDS content in the breads was 29.12%–31.32%. Previous studies about different bread types in South and Central Asia reported that the RDS rate was high in bread made from white flour; it decreased and the SDS rate increased with the introduction of components such as bran and dietary fiber into breads (Ranawana & Henry, 2013). In the study on starch fractions of white, wholemeal, and rye flour‐added breads in Turkey, 68.0% fast digestible starch and 2.0% slow digestible starch were determined in wheat bran breads (Tas & El, 2000). The study about the effects of soluble and insoluble dietary fiber additions on the digestion rate of starch reported that as the soluble dietary fiber content in the samples increased, the amount of RDS decreased and the amount of SDS increased (Bae & Lee, 2014). In conclusion, the high soluble dietary fiber content of the breads resulted in high SDS and low RDS results. The ratio of rapidly digestible starch to total starch in starchy foods is defined as the starch hydrolysis index (SHI). It relatively reflects the in vitro digestion rate and rate of starch in foods, as well as the GI value determined in vivo (Englyst et al., 2003). SHI values of the breads were found between 78.16% and 80.01%. The glycemic index (GI) is a system for determining the glycemic response of foods (Wolever, 1990). However, the predictive glycemic index has been started to be used due to its determination by blood measurement, long detection time, and high detection cost. Studies have shown that a high glycemic index diet increases the risk of cardiovascular disease and increases the risk of death (Jenkins et al., 2021). It also suggests that high GI diets may be a risk factor for insomnia in postmenopausal women (Gangwisch et al., 2020). Epidemiological studies indicate that high glycemic index diets have a moderate adverse effect on the colorectal, bladder, and kidney cancers (Turati et al., 2019). Different equations have been developed to approximate the glycemic index (Goñi et al., 1997). Predictable glycemic index values of traditional Tokat breads were 82.62%–83.63%. The pGI reflects the rate of starch digestion (Cairano et al., 2020).

**FIGURE 2:**
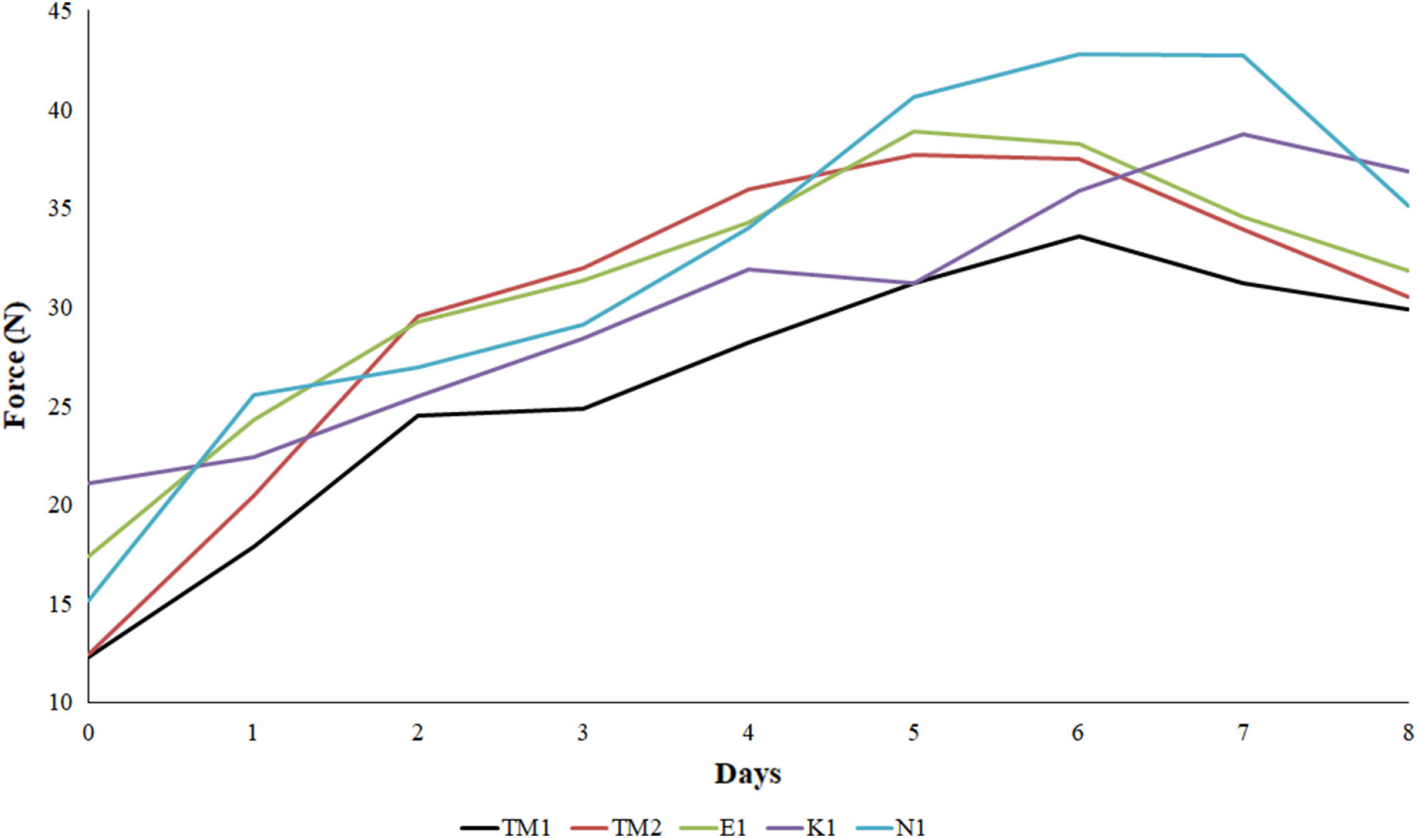
Texture analysis results of traditional Tokat bread.

The main causes of bread staling are starch retrogradation, surface drying, and binding between starch and gluten (Martin, 1991). Texture measurements during bread production and storage are important parameters used to measure bread quality and gain insight into bread staling (Lassoued et al., 2008). CO_2_ produced by yeasts and heterofermentative lactic acid bacteria, proteolysis of gluten, hydrolysis of starch, oxidation of lipids, and exopolysaccharides are effective in forming bread texture. In addition, antifungal substances produced by lactic acid bacteria in sourdough (reduction of fungus growth) and bacteriocins (reduction of pathogenic bacteria growth) are effective in the shelf life of bread (Siepmann et al., 2018). The change in the hardness of the bread was followed for 0–8 days and determined by a texture analyzer, and the results are shown in Figure 2. Day zero hardness values of the breads measured 6 h after leaving the oven varied between 12.28 and 21.08 N, with the highest hardness value found in the K1‐coded bread. The hardness values of the breads increased during storage. At the end of the sixth day, the hardness values of the breads that reached maximum hardness were determined between 33.57 and 42.77 N. Mold started on the surface of all bread samples at the end of the sixth day. TM1‐coded bread began to crumble at the end of the sixth day, TM2 and E1‐coded breads at the end of the seventh day, and K1 and N1‐coded breads at the end of the eighth day. The texture values show that traditional Tokat bread can be consumed for 5 days, provided it is kept under room conditions.

In traditional Tokat bread produced with the sourdough yeast method, in addition to normal culture yeasts, a piece of dough in which wild yeasts, lactic, acetic, and citric acid bacteria from the air, and the dough elements used are active is used as yeast in the next dough. Yeast and bacteria were isolated from 20 different sourdoughs collected from 5 different enterprises. The isolated yeast and bacteria species and their detection status in breads are shown in Table 5. Saccharomyces (6 species), Candida (3 species), Debaryomyces (1 species), Pichia (3 species), Kluyveromyces (2 species), Zygosoaccharomyces (2 species), and Torula (1 species) yeast species and Lactobacillus (6 species) and Pediococcus (2 species) bacteria were identified from 10 different sourdough samples from the Tokat region. *Saccharomyces cerevisiae*, *Debaryomyces hansenii*, *Kluyveromyces lactis*, *Lactobacillus plantarum*, and *Lactobacillus mesentorides* species were detected in all sourdough samples. In the study in which yeast characterization of sourdough samples produced in Isparta province was performed, it was reported that the dominant yeast species in the samples was *S*. *cerevisiae* (27%). *S*. *delbrueckii* (2.7%), *T*. *holmii* (10.8%), and *T*. *unisporus* (2.7%) were the other yeast species isolated (Gül et al., 2005). In the study of traditional sourdough Afyonkarahisar bread investigated, 17 different yeast species were isolated from sourdough from 25 different bakeries. *Saccharomyces cerevisiae* (68%) and *Kluyveromyces lactis* (44%) were mostly isolated in sourdoughs (Denizkara, 2021).

**TABLE 5:** Yeast and bacterial species isolated from sourdough starter.

	TM1	TM2	E1	K1	N1
*Saccharomyces cerevisiae*	+	+	+	+	+
*Saccharomyces delbrueckii*	+		+		+
*Saccharomyces dairenensis*		+			
*Saccharomyces barnetti*	+	+		+	+
*Saccharomyces uvarum*		+		+	
*Saccharomyces exiguus*	+		+	+	+
*Candida humulis*					+
*Candida krusei*		+		+	
*Candida valida*			+		
*Debaryomyces hansenii*	+	+	+	+	+
*Pichia angusta*			+		+
*Pichia cactohpila*		+			
*Pichia polymorpha*	+	+			+
*Kluyveromyces marxianus*		+	+		
*Kluyveromyces lactis*	+	+	+	+	+
*Zygosoaccharomyces lactis*	+	+			+
*Torula lactis*			+		
*Zygosaccharomyces rouxii*		+	+	+	+
*Lactobacillus brevis*	+	+	+	+	
*Lactobacillus amylophilus*		+		+	+
*Lactobacillus plantarum*	+	+	+	+	+
*Lactobacillus mesentorides*	+	+	+	+	+
*Lactobacillus casei*	+		+	+	+
*Lactobacillus acidophilus*	+	+		+	+
*Pediococcus acidilactici*	+		+	+	
*Pediococcus pentosaceus*	+	+			+

The flavor and aroma of breads are produced by volatile substances. Many factors affect these volatiles, such as raw materials, yeast components, different strains, and fermentation conditions (Kim et al., 2009; Liu et al., 2018; Xu et al., 2020; Yan et al., 2019). Sourdough is the most critical factor for volatile compounds, while wood‐fired baking is a crucial step affecting the flavor of bread (Liu et al., 2018; Xu et al., 2020; Yan et al., 2019). Lactic acid bacteria synthesize exo‐polysaccharides, which increase the acidity of sourdough, improve the structure of the dough, slow down staling, and increase digestibility (Behera & Ray, 2015). Sourdough yeasts are essential for the production of CO_2_, which leavens the dough, and for the production of flavor compounds, which, together with acids, make for a well‐balanced bread flavor. Yeasts present in sourdough are generally acid‐tolerant (Suchintita et al., 2023). Furthermore, lactic acid bacteria increase the concentration of aliphatic, dicarboxylic, and hydroxyl amino acid groups. These substances are utilized by yeasts during the fermentation phase (Minervini et al., 2014). The volatile organic compounds of breads were characterized by the presence of 45 different compounds (Table 6) belonging to six classes of compounds: alkanes, aldehydes, aromatics, acids, pyrazines, and terpenoids. It was determined that the breads contained 97.52–115.04 μg/kg ƩAlkanes, 45.76–50.19 μg/kg ƩAldehydes, 110.79–117.07 μg/kg ƩAromatic, 55.25–59.14 μg/kg ƩAcids, 5.64–6.08 μg/kg ƩPyrazines, and 3.10–3.56 μg/kg ƩTerpenoids. The incense and resinous compounds from the fire of pelite (*Quercus petraea* Lieb.) and hornbeam (*Fagus orientalis* Lipsky) used in the baking of traditional Tokat bread give Tokat bread its unique flavor. In addition, the yeast and bacterial population in sourdough also has an effect on taste and aroma. While yeasts and heterofermentative LABs in sourdough make the dough rise, homofermentative LABs affect the acidity and flavor of the bread.

**TABLE 6:** Volatile organic compounds emitted from breads.

LRI	Compounds	TM1	TM2	E1	K1	N1	Identification
	ƩAlkanes	**107.38**	**97.52**	**115.04**	**105.98**	**102.41**	
600	*n*‐Hexane	5.66 ± 0.12	5.12 ± 0.18	6.23 ± 0.08	4.38 ± 0.16	5.47 ± 0.11	MS, LRI
900	*Nonane*	*ND*	*ND*	*ND*	*ND*	*ND*	
1000	*Decane*	6.27 ± 0.20	5.36 ± 0.08	7.08 ± 0.24	6.51 ± 0.14	6.47 ± 0.16	
1025	Limonene	*ND*	*ND*	*ND*	*ND*	*ND*	
1100	Undecane	23.16 ± 1.10	18.78 ± 1.30	26.14 ± 2.11	21.63 ± 1.80	19.85 ± 1.45	
1200	Dodecane	31.45 ± 2.80	30.18 ± 2.40	33.06 ± 3.05	32.14 ± 2.88	30.43 ± 3.01	
1300	Tridecane	32.66 ± 2.84	30.12 ± 2.10	34.25 ± 1.16	33.14 ± 1.05	31.78 ± 2.30	
1400	Tetradecane	1.12 ± 0.04	0.89 ± 0.06	0.96 ± 0.02	1.04 ± 0.06	0.78 ± 0.04	
1500	Pentadecane	*ND*	0.06 ± 0.02	*ND*	*ND*	*ND*	
1600	Hexadecane	4.12 ± 0.56	3.98 ± 0.31	4.24 ± 0.12	4.08 ± 1.01	4.16 ± 0.76	
1700	Heptadecane	*ND*	*ND*	*ND*	0.02 ± 0.01	*ND*	
1800	Octadecane	0.12 ± 0.01	*ND*	0.06 ± 0.01	0.10 ± 0.01	0.07 ± 0.01	
1900	Nonadecane	*ND*	*ND*	*ND*	*ND*	*ND*	
2000	Eicosane	2.87 ± 0.20	3.11 ± 0.24	3.02 ± 0.18	2.94 ± 0.11	3.40 ± 0.32	
	ƩAldehydes	**47.80**	**46.63**	**45.76**	**50.19**	**48.47**	
801	Hexanal	11.95 ± 0.80	9.97 ± 0.54	10.78 ± 1.02	12.12 ± 1.10	11.41 ± 0.90	MS, LRI
901	Heptanal	9.34 ± 0.60	10.14 ± 0.18	9.83 ± 0.26	11.01 ± 0.80	10.49 ± 0.45	
951	(E)‐2‐Heptanal	2.38 ± 0.08	2.14 ± 0.12	2.30 ± 0.16	2.41 ± 0.18	2.26 ± 0.14	
1001	Octanal	2.18 ± 0.10	1.99 ± 0.10	1.96 ± 0.18	2.04 ± 0.12	2.21 ± 0.14	
1102	Nonanal	6.75 ± 0.40	6.82 ± 0.52	6.14 ± 0.38	7.04 ± 0.50	6.84 ± 0.24	
1160	(E)‐2‐Nonanal	9.27 ± 0.44	9.56 ± 0.48	8.97 ± 0.34	9.43 ± 0.38	9.17 ± 0.21	
1205	Decanal	5.93 ± 0.06	6.01 ± 0.08	5.78 ± 0.10	6.14 ± 0.10	6.09 ± 0.12	
	ƩAromatic	**110.79**	**115.80**	**113.16**	**117.07**	**114.34**	
833	Furfural	3.56 ± 0.12	3.45 ± 0.18	3.60 ± 0.14	3.28 ± 0.04	3.52 ± 0.09	MS, LRI
837	3‐Hydroxymethylfuran	0.87 ± 0.02	1.01 ± 0.05	0.94 ± 0.04	0.97 ± 0.06	1.03 ± 0.05	
852	Ethylbenzene	0.26 ± 0.01	0.12 ± 0.01	*ND*	0.20 ± 0.01	0.18 ± 0.01	
860	1,3‐Dimethylbenzene	1.95 ± 0.10	2.02 ± 0.20	2.11 ± 0.18	1.98 ± 0.07	2.06 ± 0.11	
888	Styrene	1.66 ± 0.33	1.72 ± 0.21	1.60 ± 0.24	1.74 ± 0.14	1.65 ± 0.16	
909	2‐Acetylfuran	*ND*	*ND*	*ND*	0.04 ± 0.01	*ND*	
952	Benzaldehyde	86.25 ± 6.78	91.14 ± 8.11	88.63 ± 7.33	92.40 ± 8.74	89.73 ± 7.47	
964	1,3,5‐Trimethylbenzene	0.38 ± 0.04	0.40 ± 0.02	0.35 ± 0.03	0.34 ± 0.02	0.38 ± 0.06	
1022	*p*‐Cymene	3.17 ± 0.21	2.96 ± 0.18	3.21 ± 0.11	3.11 ± 0.14	2.94 ± 0.18	
1036	Benzeneacetaldehyde	12.69 ± 0.08	12.98 ± 0.10	12.72 ± 0.11	13.01 ± 0.06	12.85 ± 0.07	
	ƩAcids	**56.07**	**55.25**	**59.14**	**57.56**	**56.64**	
1418	Acetic acid	51.23 ± 2.13	50.23 ± 1.87	54.17 ± 2.02	52.46 ± 1.92	51.64 ± 1.90	MS, LRI
1508	Propanoic acid	*ND*	*ND*	0.03 ± 0.01	*ND*	0.04 ± 0.01	
1750	Pentanoic acid	4.84 ± 0.13	5.02 ± 0.22	4.94 ± 0.18	5.10 ± 0.18	4.96 ± 0.14	
	ƩPyrazines	**6.08**	**5.64**	**5.86**	**5.72**	**6.04**	
821	2‐Methyl‐pyrazine	0.72 ± 0.02	0.56 ± 0.04	0.61 ± 0.04	0.58 ± 0.03	0.70 ± 0.06	MS, LRI
865	Ethyl‐pyrazine	1.16 ± 0.03	0.98 ± 0.04	1.06 ± 0.03	1.02 ± 0.03	1.20 ± 0.05	
982	2‐Ethyl‐3‐methylpyrazine	2.18 ± 0.31	2.06 ± 0.28	2.22 ± 0.18	2.16 ± 0.21	2.08 ± 0.11	
1044	2,3‐dimethyl‐5‐ethylpyrazine	2.02 ± 0.27	2.04 ± 0.22	1.97 ± 0.24	1.96 ± 0.26	2.06 ± 0.21	
	ƩTerpenoids	**3.47**	**3.10**	**3.56**	**3.37**	**3.41**	
928	*α*‐Pinene	0.18 ± 0.01	ND	0.12 ± 0.01	0.16 ± 0.01	0.08 ± 0.00	MS, LRI
981	*β*‐Pinene	2.02 ± 0.13	1.88 ± 0.14	2.11 ± 0.12	1.94 ± 0.15	2.06 ± 0.21	
993	*α*‐Terpinene	0.48 ± 0.02	0.44 ± 0.05	0.51 ± 0.03	0.49 ± 0.04	0.41 ± 0.04	
1038	*β*‐(*Z*)‐Ocimene	*ND*	*ND*	*ND*	*ND*	*ND*	
1040	*β*‐Ocimene	0.79 ± 0.08	0.78 ± 0.10	0.82 ± 0.11	0.74 ± 0.09	0.86 ± 0.12	
1057	*γ*‐Terpinene	*ND*	*ND*	*ND*	0.04 ± 0.01	*ND*	
1066	*α*‐Terpinolene	*ND*	*ND*	*ND*	*ND*	*ND*	

*Note*: Results are expressed in μg/kg. Components listed in order of elution on a DB‐5MS and DB‐WAX column. Compounds divided into different chemical classes (alkanes, aldehydes, aromatic, acids, pyrazines, and terpenoids) are classified in order of LRI (Linear Retention Index) of apolar column (DB‐5MS). Bold values indicate the total values of the related heading. The different letters indicate statistical differences at the *p* < .05 level of the samples in the same line.

Abbreviation: ND, not detected.

## 4 CONCLUSIONS

This study was carried out to standardize the production of traditional Tokat bread, which does not have standard production conditions, and to determine its characteristics. The characteristics of traditional Tokat bread, which is consumed with its unique texture and aroma, were determined. Traditional Tokat bread, which has a longer shelf life than Turkish‐type francala bread, stands out with its thick crust structure, frequent pores, large size, and intense aroma. Sourdoughs used in its production and 45 different flavoring substances formed by the wood used in baking were identified. This study will guide future research on traditional breads and fill the gap in the literature. The study also provides useful information for the standard commercial production of traditional Tokat bread. In addition, this study is a source for books, almanacs, etc. to be prepared about breads.

## AUTHOR CONTRIBUTIONS

**Ali Cingöz:** Investigation (equal); methodology (equal); supervision (equal); visualization (equal); writing – original draft (equal); writing – review and editing (equal).

## CONFLICT OF INTEREST STATEMENT

The authors are do not having any conflicts of interest related to the manuscript.

## ETHICS STATEMENT

This study does not involve any human or animal testing.

## Data Availability

Data will be made available on request.
